# Clinical pathways of hospitalized extremity soft-tissue infections: a retrospective study

**DOI:** 10.1007/s00423-026-04287-w

**Published:** 2026-10-02

**Authors:** Jonas Roos, Antonia Krause, Christian Prangenberg, Alberto Alfieri Zellner, Franziska Kappenberg, Matthias Schmid, Kristian Welle, Thorsten Hornung

**Affiliations:** 1 University Hospital Bonn, Department of Orthopedics and Trauma Surgery, University of Bonn, Venusberg-Campus 1, Bonn, 53127 Germany; 2 University Hospital Bonn, Institute for Medical Biometry, Informatics and Epidemiology, University of Bonn, Bonn, Germany; 3 University Hospital Bonn, Medical Management Division, University of Bonn, Bonn, Germany; 4 University Hospital Bonn, Department of Dermatology and Allergology, University of Bonn, Bonn, Germany

**Keywords:** Skin and soft-tissue infection, Extremity infections, Cellulitis, Erysipelas, Abscess, Computed tomography, Ultrasonography

## Abstract

**Background:**

Bacterial infections of the skin and soft tissues (SSTIs) are common and should be considered a key differential diagnosis in patients presenting with redness and swelling in these areas. They range from mild superficial cases to severe, complicated forms requiring prompt antimicrobial therapy and, often, surgical intervention. We aimed to describe clinical pathways, diagnostic evolution, treatment strategies, and outcomes in a hospitalized orthopedic/trauma surgery cohort.

**Methods:**

We performed a retrospective single-center analysis of all inpatients treated for extremity SSTIs between January 2020 and December 2023. Extracted variables included demographics, admission and discharge diagnoses, inflammatory markers, imaging, microbiology, surgical procedures, antibiotic regimens and duration, intensive care unit (ICU) utilization, and in-hospital outcomes.

**Results:**

A total of 242 patients were included (mean age 55.7 ± 20.3 years; 40.1% female), with a median length of stay of 7.5 days. ICU care was required in 25/242 10.3%; in-hospital mortality was 7/242 2.9%. Surgical management was required in 134/242 patients (55.4%). Comorbidity burden was substantial (ASA III–IV: 41.3%), and sepsis was documented in 32/242 (13.2%). Diagnostic classification changed between admission and discharge in 80/242 cases (33.1%) of cases. Inflammatory markers declined markedly from admission to discharge (CRP 43.0 [Range: 0.6–640.2, IQR: 13.2–107.5] to CRP: 13.1 [Range: 0.4–541.0, IQR: 4.6–35.2]; leukocytes 11.9 ± 6.09 10/L to 7.68 ± 4.25 10^9^/L). The hand, lower leg and foot were the most frequent sites. CT was performed in 111/242 (45.9%) and ultrasound in 65/242 (26.9%), with heterogeneous imaging strategies (CT only 87/242 (36.0%), US only 41/242 (16.9%), both 24/242 (9.9%), none 90/242 (37.2%)). CT use clustered with higher admission CRP and higher operative burden, including more re-interventions among operated patients. Cultures were frequently negative; when positive, staphylococci and streptococci predominated. Ampicillin/sulbactam and amoxicillin/clavulanate were the most common antibiotics, with frequent IV combination therapy.

**Conclusions:**

Hospitalized extremity SSTIs were characterized by frequent diagnostic reclassification, heterogeneous documentation and imaging pathways, and a high rate of operative management despite overall favorable outcomes. CT was more frequently used in patients with higher inflammatory activity and greater procedural burden. This descriptive association may, at least in part, reflect differences in clinical severity and selection for imaging. The findings support structured reassessment and targeted imaging when anatomical clarification is likely to alter management.

## Introduction

Bacterial infections of the skin and soft tissues (SSTI) are common and should be considered a key differential diagnosis in patients with redness and swelling in these areas [1]. They range from mild, superficial cases to severe, complicated forms requiring prompt antimicrobial and often surgical intervention [2].

This broad clinical group is classified into distinct entities based on anatomical depth of involvement and severity. Erysipelas is a superficial cutaneous infection affecting the epidermis and superficial dermis with prominent lymphatic involvement, characterized by sharply demarcated erythematous plaques that are raised above the surrounding skin, with characteristic tongue-shaped extensions [3]. It is predominantly caused by β-hemolytic streptococci, primarily group A streptococcus (*Streptococcus pyogenes*) [4, 5]. A Slovenian study reported erysipelas as a common dermatological disease, with an annual incidence of 113 cases per 100,000 population. It predominantly affects older adults and occurs more often in women than in men [6, 7].

Cellulitis is a non-purulent, non-loculated infection of the deeper dermis and subcutaneous tissue that does not extend to the fascia. It is clinically characterized by poorly demarcated, rapidly advancing erythematous lesions with associated edema and lymphangitic involvement [8]. Unlike erysipelas, cellulitis involves both β*-hemolytic streptococci* and *Staphylococcus aureus*, with the latter particularly prevalent in traumatic or surgical contexts [9, 10]. Limited cellulitis presents without systemic toxicity and without uncontrolled comorbidities, typically amenable to outpatient oral antimicrobial therapy with localized erythema [11]. Severe cellulitis is characterized by signs of marked systemic illness [12]. It carries risk of progression to deeper tissue involvement and sepsis [13].

This historical classification has been applied locally during the treatment period. However, current guidelines are under revision because these terms—particularly in German medical literature—have been used interchangeably, thereby obscuring critical distinctions in severity and tissue involvement, and hindering appropriate antimicrobial targeting and clinical risk stratification [5, 14].

SSTI often present diagnostic challenges, as distinguishing between types and selecting appropriate treatment can be difficult in the absence of clear signs of an abscess [15]. Ultrasonography (US) can help rule out non-infectious causes of swelling, such as deep vein thrombosis, and influences the treatment decision for cellulitis [15]. Furthermore, the quality of the findings are usually dependent on the experience of the examiner. Computed tomography (CT), by contrast, provides detailed anatomical views and is highly sensitive for detecting soft-tissue gas [16].

The aim of the study was to describe the clinical characteristics, diagnostic pathways, therapeutic strategies, and outcomes of patients with skin and soft tissue infections, and to investigate associations between comorbidities, infection markers, diagnostic imaging, surgical interventions, antibiotic therapy, and patient outcomes.

## Methods

### Study design

A retrospective analysis of patient volume in orthopedics and trauma surgery (O/T) at a university maximum care provider was conducted between January 2020 and December 2023. The study was approved by the local Institutional Review Board (approval no. 406/17).

### Inclusion and exclusion criteria

All patients who were treated as inpatients for soft tissue infections between January 2020 and December 2023 were considered eligible for this study, including patients with erysipelas, cellulitis, and abscesses, as well as patients with infections following animal bites. Patients who were treated on an outpatient basis or in other specialist departments were excluded. Patients with implants for whom a soft-tissue infection was the primary indication for treatment were not excluded. No additional exclusion criterion was applied based on immunosuppression, chronic corticosteroid therapy, diabetes mellitus, or other comorbidities.

### Data extraction / collected variables

Clinical and administrative data were extracted from the local electronic data management system (ORBIS^®^, Dedalus Group, Mortsel, Belgium) and entered into a spreadsheet for analysis. No retrospective study-specific diagnostic adjudication was performed. Consequently, changes between admission and discharge categories represent changes in the documented clinical diagnosis during the course of hospitalization and may reflect diagnostic clarification, disease evolution, or differences in clinical terminology. Extracted variables comprised patient characteristics (sex and age), admission and discharge dates with calculated length of stay, infection characteristics (anatomical localisation, admission diagnosis, signs of sepsis and clinical demarcation), discharge diagnosis, imaging diagnostics (CT and US), microbiological findings (pathogen detection), laboratory parameters at admission and discharge (C-reactive protein and leukocyte count), therapeutic steps (diagnostic work-up, initiation of antibiotic therapy, and surgical intervention), procedural metrics (number of operations and revision procedures), antibiotic regimen and duration (oral, intravenous, and total), outcome, perioperative risk classification (ASA score), intensive care unit (ICU) admission and duration. 

### Statistical analysis

Continuous data are described using means with standard deviations (SD) or medians with ranges and interquartile ranges (IQR). Categorical data are described using frequency distributions with percentages for categorical variables.

All analyses were carried out using the R Software for Statistical Computation, Version 4.5.0 [17], and visualizations were created using the software packages ggplot2 [18], ggalluvial [19], eulerr [20].

### Declaration of generative AI and AI-assisted technologies in the writing process

GPT-5.2 was used for language improvement and general manuscript revision. After using this tool, the authors reviewed and edited the content as needed and take full responsibility for the publication’s content.

## Results

A total of 242 patients (mean age 55.7 ± 20.3 years (median 57, IQR (40.2–73)), 40.1% females) were included in the study. The median length of stay was 7.5 [1–154, IQR 5–18] days. Overall, 134 patients (55.4%) underwent surgery, while 108 (44.6%) were managed without an operation. ASA physical status was distributed as follows: ASA I in 64 patients (26.4%), ASA II in 78 (32.2%), ASA III in 88 (36.4%), and ASA IV in 12 (5.0%). Patients classified as ASA III–IV had a substantial burden of clinically relevant comorbidities. Diabetes mellitus was documented in 39/100 (39.0%) and renal insufficiency, nephropathy, or relevant renal failure in 27/100 (27.0%). Peripheral arterial disease was present in 8/100 (8.0%), lymphedema in 3/100 (3.0%), and confirmed chronic venous insufficiency in 1/100 (1.0%). Conditions or treatments potentially affecting immune competence were documented in 9/100 (9.0%) patients. Signs of sepsis were documented in 32 patients (13.2%), absent in 186 (76.9%), and unclear in 24 (9.9%). Clinical demarcation was described as diffuse in 115 cases (47.5%) and smoothly demarcated in 4 cases (1.7%); it was unclear in 121 cases (50.0%) and missing in 2 cases (0.8%). An ICU stay was required in 25 patients (10.3%). Among ICU-treated patients, 18 had one ICU admission, 5 had two, and 2 had three ICU admissions. Overall ICU length of stay was a median of 5 [1–94, IQR: 2–20] days per patient, with lengths of stays added up for several admissions where applicable. Overall outcomes were favorable: 214 (88.4%) were discharged as healed, 12 (5.0%) suffered a relapse after discharge, 7 (2.9%) died, and 9 (3.7%) were referred for palliative medical continuation of care. Throughout this section, n refers to the number of patients.

**Fig. 1:**
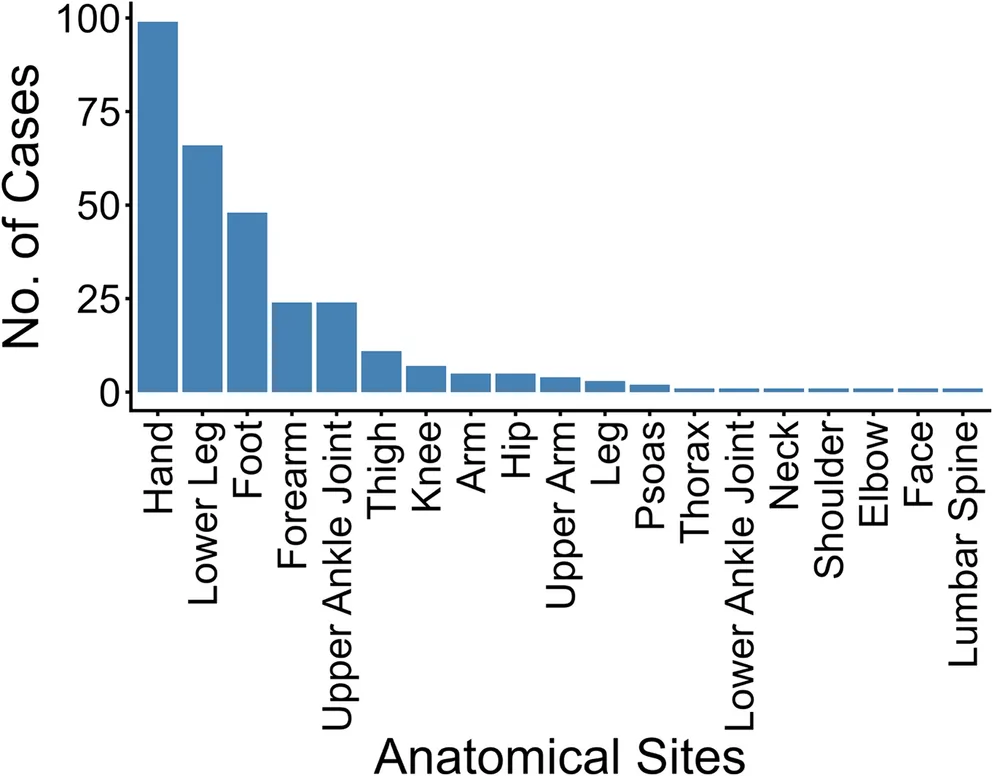
Distribution of infection localizations in the study cohort. Bars indicate the absolute number of cases per anatomical site, with the hand, lower leg, and foot representing the most frequent localizations

A total of 323 anatomical sites were documented. Most patients had involvement of one site (*n* = 184), followed by two sites (*n* = 41), three sites (*n* = 11), and four sites (*n* = 6), corresponding to 242 cases in total. The hand (*n* = 99), lower leg (*n* = 66), foot (*n* = 48), and forearm (*n* = 24) were the main areas affected. The most common combinations of two body parts were foot and lower leg (*n* = 12), hand and forearm (*n* = 12), and hand and lower leg (*n* = 5). Less common combinations were forearm and lower leg (*n* = 4), foot and hand (*n* = 3), lower leg and knee (*n* = 3), and all other combinations (*n* ≤ 2 each). Figure 1 shows a distribution across the various anatomical locations, whereby a patient could also have multiple locations.

### Diagnosis

**Fig. 2:**
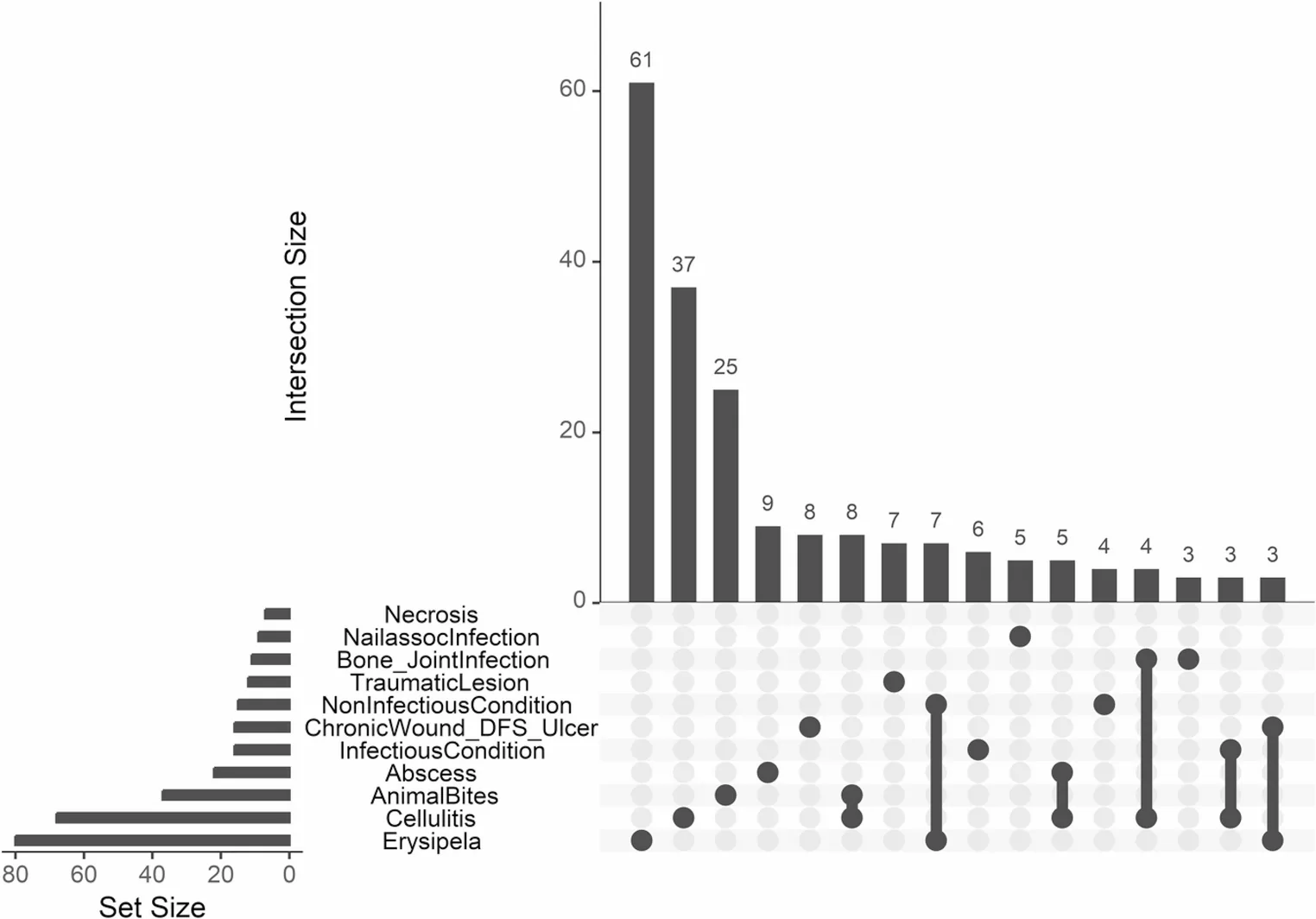
Set sizes (left horizontal bars) show the total frequency of each admission diagnosis across all cases. Intersection sizes (top vertical bars) depict the frequency of single diagnoses and diagnostic combinations (connected dots) occurring more than once. Erysipelas and cellulitis constituted the largest single-diagnosis groups (*n* = 61 and *n* = 37, respectively), followed by animal bites (*n* = 25). The most frequent co-diagnoses were animal bite with cellulitis (*n* = 8) and abscess with cellulitis (*n* = 5). Suspected diagnoses were included, and multiple admission diagnoses per case were possible

Across all admission diagnoses (allowing for multiple diagnoses per case), erysipelas was most common (*n* = 80), followed by cellulitis (*n* = 68), animal bites (*n* = 37), abscesses (*n* = 22), and chronic wounds/diabetic foot syndrome/ulcers (*n* = 16). Further diagnoses included other infectious conditions (*n* = 16), other non-infectious conditions (*n* = 15), traumatic lesions (*n* = 12), bone and joint infections (*n* = 11), and nail-associated infections (*n* = 9), while no identifiable diagnosis was documented in 17 cases; necrosis (*n* = 7). Suspected diagnoses were included in these counts, and combinations of admission diagnoses were possible (Fig. 2).

Restricting the analysis to diagnoses and diagnostic combinations occurring more than once, isolated erysipelas predominated (*n* = 61), followed by isolated cellulitis (*n* = 37) and isolated animal bite (*n* = 25). Recurrent additional single diagnoses were abscess (*n* = 9) and chronic wounds/ulcers (*n* = 8), as well as traumatic lesions (*n* = 7), nail-associated infections (*n* = 5), and other infectious (*n* = 6) or other non-infectious conditions (*n* = 4). The most frequent co-diagnoses were animal bite with cellulitis (*n* = 8) and abscess with cellulitis (*n* = 5). Further recurrent combinations were chronic wounds/ulcers with erysipelas (*n* = 3), bone and joint infections (*n* = 3), and other infectious conditions plus cellulitis (*n* = 3); all remaining combinations were observed only twice (*n* = 2) each (Fig. 2).

At discharge, erysipelas (*n* = 86) and cellulitis (*n* = 84) were the most frequent diagnoses, possibly in combination with other diagnoses, followed by abscess (*n* = 34) and animal bite (*n* = 32). Among recurrent combinations, animal bite with cellulitis was most common (*n* = 8), followed by cellulitis with necrosis (*n* = 6); all other combinations occurred infrequently (≤ 4 cases each).

**Fig. 3:**
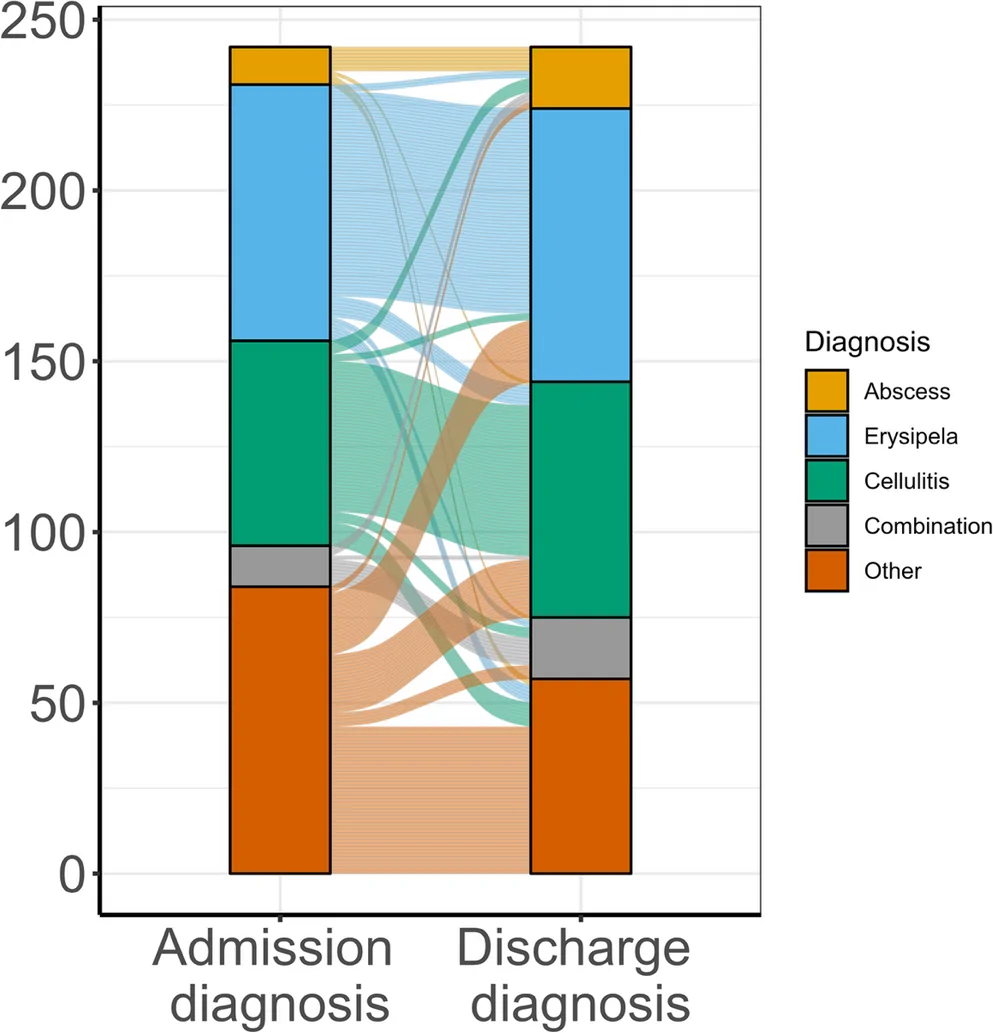
Alluvial plot depicting transitions between admission and discharge diagnosis categories in the study cohort (*n* = 242). Diagnosis groups were defined as follows: patients with exactly one of the target diagnoses—abscess, erysipelas, or cellulitis —were assigned to the corresponding category irrespective of concomitant diagnoses; patients with two target diagnoses were classified as “Combination”; all remaining cases were grouped as “Other.” Stacked bars represent the distribution of diagnostic categories at admission and discharge, and connecting flows indicate patient-level changes between categories; flow width is proportional to the number of cases

Across the 242 cases, the admission and discharge categories were concordant in 162 patients (66.9%) and changed in 80 patients (33.1%). Figure 3 provides an overview of the process. The most frequent reclassifications were from “other” at admission to erysipelas (*n* = 18) or to cellulitis (*n* = 17). Additional, less common shifts included cellulitis to “other” (*n* = 7) and erysipelas to cellulitis (*n* = 6); all remaining transitions occurred infrequently.

### Diagnostics

The median CRP at admission was 42.95 [Range: 0.60–640.21, IQR: 13.19-107.46] and at discharge 13.07 [Range: 0.43–540.96, IQR: 4.58–35.17]. Three values were missing at admission and 19 at discharge. The mean leukocyte count at admission was 11.88 ± 6 10^9^/L and at discharge 7.68 ± 4.2 10^9^/L. Four values were missing at admission and 19 at discharge. These trends were consistent across diagnostic subgroups (abscess, erysipelas, cellulitis, combined presentations, and other diagnoses), with higher admission CRP and leukocyte counts particularly in abscess/cellulitis and combined cases and a general decline in both markers by discharge (Table 1).

**Table 1:** Admission and discharge inflammatory markers (CRP and leukocyte count) stratified by admission diagnosis (abscess, erysipelas, cellulitis, combined, other, overall)

	Abscess	Erysipela	Cellulitis	Combination	Other	Overall
	(*N* = 11)	(*N* = 75)	(*N* = 60)	(*N* = 12)	(*N* = 84)	(*N* = 242)
CRP at admission						
Mean (SD)	109 (99.6)	86.6 (102)	106 (111)	130 (109)	50.8 (69.7)	82.2 (97.5)
Median [Min, Max]	85.3 [5.75, 346]	50.2 [0.860, 640]	55.4 [0.600, 377]	128 [5.36, 287]	21.8 [0.600, 304]	43.0 [0.600, 640]
Q1 - Q3	47.6–131	25.5–102	18.2–167	13.3–224	4.91–53.0	13.2–107
Missing	0 (0%)	0 (0%)	1 (1.7%)	0 (0%)	2 (2.4%)	3 (1.2%)
CRP at discharge						
Mean (SD)	33.7 (32.4)	28.8 (33.7)	28.2 (43.0)	14.7 (16.4)	26.0 (64.2)	27.2 (47.3)
Median [Min, Max]	22.8 [0.920, 89.3]	17.5 [0.600, 172]	9.32 [0.600, 237]	6.52 [1.40, 50.2]	10.4 [0.430, 541]	13.1 [0.430, 541]
Q1 - Q3	11.7–46.5	5.88–36.4	3.88–36.9	3.65–25.8	3.71–27.8	4.58–35.2
Missing	1 (9.1%)	2 (2.7%)	6 (10.0%)	0 (0%)	10 (11.9%)	19 (7.9%)
Leukocyte count at admission						
Mean (SD)	15.8 (7.48)	11.3 (5.03)	14.4 (7.41)	14.5 (10.5)	9.73 (3.54)	11.9 (6.09)
Median [Min, Max]	16.7 [5.25, 27.5]	10.2 [4.15, 30.3]	12.7 [4.22, 49.9]	11.4 [7.12, 43.3]	9.18 [0.160, 21.5]	10.4 [0.160, 49.9]
Q1 - Q3	9.45–19.5	7.95–12.6	9.40–17.0	8.50–13.8	7.56–11.3	8.07–14.4
Missing	0 (0%)	1 (1.3%)	2 (3.3%)	0 (0%)	1 (1.2%)	4 (1.7%)
Leukocyte count at discharge						
Mean (SD)	13.0 (12.7)	7.68 (3.88)	7.28 (3.20)	8.13 (4.32)	7.18 (2.42)	7.68 (4.25)
Median [Min, Max]	8.73 [5.14, 47.5]	6.53 [3.11, 23.6]	6.55 [3.36, 24.9]	6.53 [2.38, 18.2]	6.72 [3.25, 18.7]	6.65 [2.38, 47.5]
Q1 - Q3	6.69–12.6	5.08–8.64	5.52–7.76	5.69–9.89	5.77–8.01	5.52–8.59
Missing	1 (9.1%)	2 (2.7%)	6 (10.0%)	0 (0%)	10 (11.9%)	19 (7.9%)

### Microbiological spectrum

**Fig. 4:**
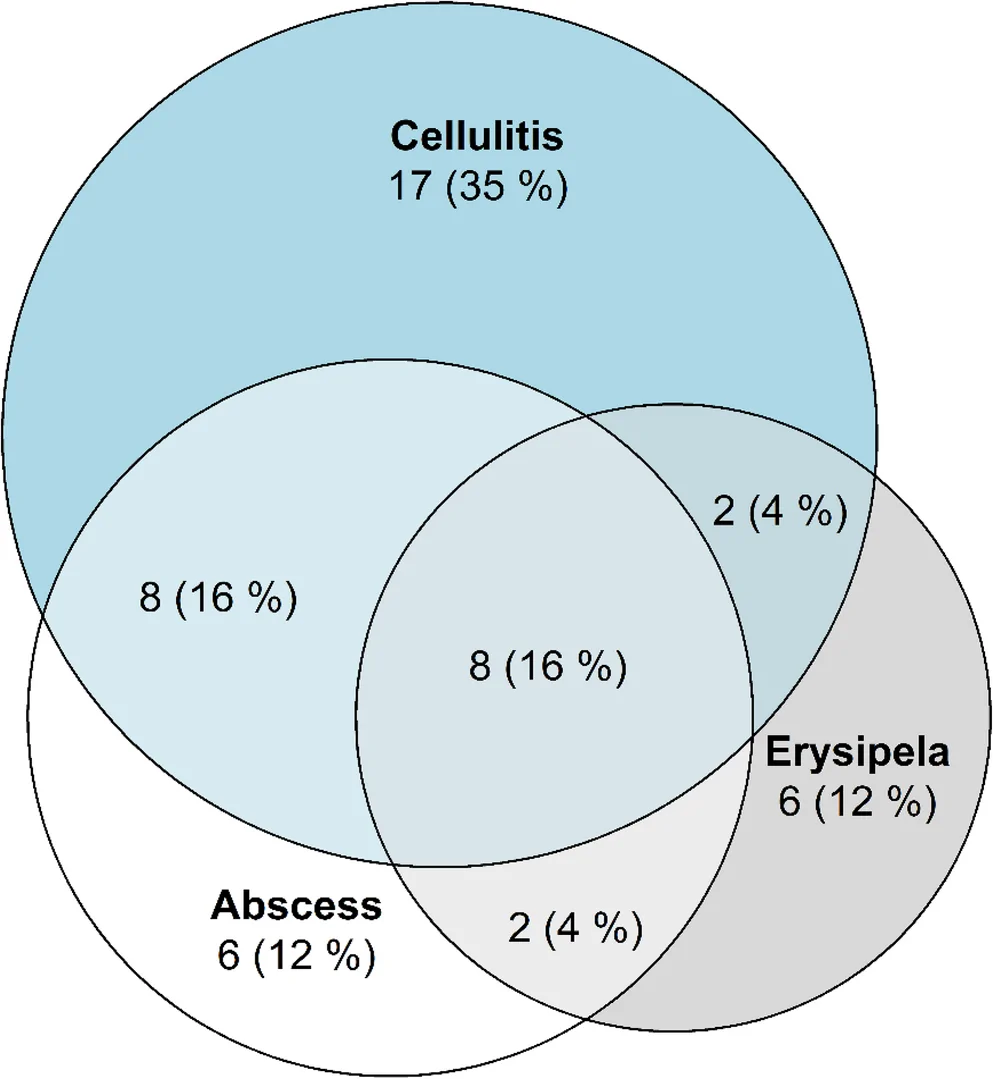
Venn diagram illustrating overlap between the diagnostic groups abscess, erysipelas, and cellulitis in the diagnosis-stratified analysis of pathogen detection. Numbers (with percentages) indicate the number of cases assigned exclusively to a single diagnosis group or concurrently to multiple groups (intersection areas). For these stratified analyses, no distinction was made between isolated and combined diagnoses; consequently, patients with more than one of the target diagnoses were counted in each corresponding group, accounting for parts of the observed overlap

*Staphylococcus aureus* was the most common organism (*n* = 27), followed by *Staphylococcus epidermidis* (*n* = 14) and *Streptococcus pyogenes* (*n* = 9). Further recurrent isolates included *Streptococcus dysgalactiae* (*n* = 7), *Enterococcus faecalis* and *Streptococcus agalactiae* (each *n* = 6), *Pseudomonas aeruginosa* (*n* = 5), *Enterobacter cloacae* (*n* = 4), and several other Gram-negative organisms at lower frequencies (e.g., *Escherichia coli*, *Pasteurella multocida*). In cases including abscess, cultures were negative in 7; when positive, *Staphylococcus aureus* (*n* = 7) and *Staphylococcus epidermidis* (*n* = 6) predominated. In cases including cellulitis, cultures were negative in 38, with *S. aureus* (*n* = 10) the most frequent isolate, followed by *Streptococcus agalactiae* (*n* = 5). In cases including erysipelas, cultures were negative in 62 and, when positive, most commonly yielded *S. aureus* (*n* = 10); other organisms were only sporadically detected. The overlap between abscess, erysipelas, and cellulitis in this diagnosis-stratified pathogen analysis is illustrated in Fig. 4, highlighting that combined presentations were counted in each respective category.

### Antibiotic therapy

Antibiotic therapy was a central component of inpatient management. Overall, the most frequently prescribed agents were ampicillin/sulbactam (ampicillin/sulbactam; *n* = 135), amoxicillin/clavulanate (*n* = 82), piperacillin/tazobactam (*n* = 62), cefuroxime (*n* = 54), clindamycin (*n* = 50), and metronidazole (*n* = 48). When stratified by route, oral regimens were dominated by amoxicillin/clavulanate (*n* = 82) and clindamycin or ampicillin/sulbactam (each *n* = 31), whereas intravenous therapy most commonly included ampicillin/sulbactam (*n* = 131), followed by piperacillin/tazobactam (*n* = 62), cefuroxime (*n* = 53), metronidazole (*n* = 46), and clindamycin (*n* = 38). Most patients received one oral antibiotic (152/242), while 37/242 received ≥ 2 oral agents; for intravenous therapy, 120/242 received one IV antibiotic and 113/242 received ≥ 2 IV agents. The cumulative duration of oral antibiotic exposure per patient had a median of 11 days (IQR 8–15; mean 12.4; range 1–45), while IV exposure had a median of 8 days (IQR 6–17; mean 14; range 1–123). Patients received antibiotics for a median duration of 16 days (IQR 13–27; mean 21.7; range 1–123).

### Imaging

CT was performed in 111 patients (45.9%) and US in 65 (26.9%); 90 (37.2%) received no imaging, 87 (36.0%) CT only, 41 (16.9%) US only, and 24 (9.9%) both modalities.

When stratified by imaging modality, surgery was slightly more frequent among patients who underwent CT than among those who did not (58.6% vs. 52.7%).

Conversely, when stratified by treatment group, ultrasound was documented more often in non-operated than in operated patients (41.7% vs. 14.9%). By imaging category, the surgical rate was 67.8% for CT only, 61.1% for no imaging, 34.1% for ultrasound only, and 25.0% for both modalities. Among operated patients, those with CT more often required multiple procedures (≥ 4 operations: 32.3% vs. 11.6%; maximum 11 vs. 7). Admission inflammatory markers differed by imaging strategy: CRP was highest in the CT group (mean 116 ± 117 mg/L; median 72.3 mg/L [1.01–640]) compared with no imaging (59.2 ± 66.8; median 37.9 [0.60–278]) and US only (37.7 ± 52.9; median 14.3 [1.68–232]). Leukocyte counts showed a similar, though less pronounced pattern (CT: 13.1 ± 6.59 10^9^/L; median 11.4 10^9^/L [0.16–43.3 10^9^/L] vs. no imaging: 10.6 ± 6.04 10^9^/L; median 9.08 10^9^/L [3.73–49.9 10^9^/L] and US only: 11.3 ± 3.8 10^9^/L; median 10.2 10^9^/L [6.42–22.8 10^9^/L]).

### Physical status and in-hospital outcome

**Fig. 5:**
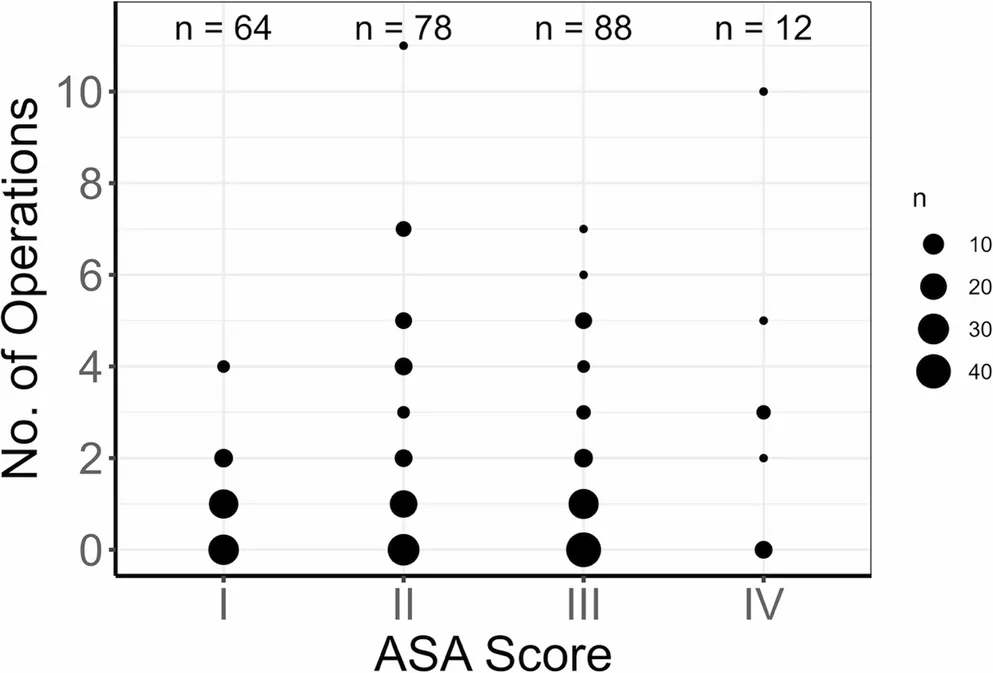
Bubble plot showing the relationship between ASA physical status class (x-axis; I–IV) and the number of surgical procedures per patient (y-axis). Each point represents an observed ASA–procedure-count combination, and bubble size is proportional to the number of patients (n) in each bubble

In-hospital deaths occurred only among patients classified as ASA III (4/88 [4.5%]) and ASA IV (3/12 [25.0%]), whereas no deaths were observed among patients classified as ASA I–II. The relationship between ASA class and the number of surgical procedures showed a broad distribution within each ASA category, with higher procedure counts also present in higher ASA classes (Fig. 5).

## Discussion

In this cohort of 242 hospitalized SSTI patients, outcomes were overall favorable, with clinical resolution in most cases and a marked decline in inflammatory markers from admission to discharge, despite a high surgical rate and substantial comorbidity burden. Diagnostic classification evolved frequently, as one-third of cases were reclassified between admission and discharge, underscoring the dynamic and initially uncertain nature of SSTI assessment. Imaging and microbiology reflected routine clinical practice, with CT used in nearly half of patients, predominantly staphylococcal/streptococcal isolates when cultures were positive, and high rates of negative cultures in erysipelas/cellulitis.

Most patients in this study were of middle age (IQR (40.2–73)), and those classified as ASA III had a substantial burden of pre-existing comorbidity. In line with this, other cohorts—particularly in cellulitis—have likewise reported a high comorbidity profile, which is consistent with prior literature describing comorbidity as a relevant risk factor [8, 21]. In this cohort, 10.3% of patients required ICU care and 2.9% died during hospitalization, while 3.7% were transitioned to palliative management. These figures are broadly consistent with earlier reports—particularly in cohorts focusing on cellulitis —supporting the interpretation that our sample is representative. Published in-hospital mortality rates for comparable soft-tissue infection cohorts have been reported in the range of approximately 1.1–3.9%, and overall ICU admission rates are generally described as low [21–24].

Most soft-tissue infections in this cohort were located in the distal extremities, particularly the hand, lower leg, and foot. This distribution is consistent with the literature and likely reflects common portals of entry, such as skin breaches from minor wounds or animal/human bites [25].

Imaging should be considered an adjunct to clinical assessment and primarily applied when anatomical clarification is expected to affect management. In our cohort, CT was performed in 45.9% of patients and was preferentially used in those with higher admission inflammatory markers, whereas ultrasound-only cases showed lower CRP levels and were more often managed non-operatively. Correspondingly, surgical treatment was somewhat more frequent in patients undergoing CT than in those without CT, and re-interventions were more common among operated patients with CT. The observed association between CT use, higher inflammatory markers, and greater procedural burden may primarily reflect confounding by indication, with CT being more frequently obtained in patients with clinically suspected severe or complex disease. Given the retrospective descriptive design and the absence of multivariable adjustment, these findings should be considered hypothesis-generating and do not establish an independent effect of CT use on operative management. In clinically uncertain cases, CT may nevertheless provide relevant anatomical information regarding occult abscess formation or deep extension that can contribute to subsequent management decisions. Especially since earlier studies report abscess rates of 8 to 36.6% in cellulitis [26–30]. Key risk factors—including diabetes mellitus, smoking, male sex, alcohol misuse, and delayed initiation of therapy—appear to play an important role [29]. When considering the appropriate diagnostic method, the literature indicates that ultrasound examination is more sensitive than CT, but CT is more specific for superficial soft tissue abscesses [31]. However, it is difficult to distinguish between simple cellulitis, which requires conservative treatment, and severe cellulitis, which requires surgical treatment. CT diagnostics can be helpful in this regard to identify complications of cellulitis and to localize the anatomical compartment affected by infection [32].

The most frequently identified pathogens were staphylococci and streptococci, followed by enterococci and *Pseudomonas spp*. This distribution is consistent with prior reports describing cellulitis as predominantly caused by Gram-positive organisms (particularly streptococci and *Staphylococcus aureus*), with Gram-negative pathogens such as *Pseudomonas spp*. and Enterobacterales/Enterobacteriaceae occurring less frequently [8, 33]. In our cohort, the predominance of Gram-positive pathogens provides a plausible rationale for the empiric regimens that were most commonly used, which largely targeted streptococci and staphylococci (e.g., aminopenicillin/β-lactamase inhibitor combinations, second-generation cephalosporins, and clindamycin). At the same time, the substantial use of broader-spectrum agents such as piperacillin/tazobactam and frequent combination IV therapy likely reflects case selection toward more severe or diagnostically uncertain infections, concern for deep/complicated disease, and the clinically relevant—though less frequent—occurrence of Gram-negative organisms including *Pseudomonas* and Enterobacterales. The overall duration of antimicrobial therapy also warrants consideration from an antimicrobial stewardship perspective. The median total treatment duration was 16 days, whereas the mean was 21.7 days, indicating a right-skewed distribution driven by a subgroup of patients receiving prolonged treatment courses. This likely reflects the selected inpatient population, including complex infections and patients requiring repeated surgical procedures. Nevertheless, the prolonged and heterogeneous treatment durations emphasize the importance of regular reassessment, microbiology-guided de-escalation, and limiting antimicrobial exposure whenever clinically appropriate.

## Conclusion

In this retrospective cohort of hospitalized patients with soft-tissue infections of the extremities, clinical outcomes were overall favorable, with substantial declines in CRP and leukocyte counts and low in-hospital mortality despite a high rate of surgical treatment and a pronounced comorbidity burden. However, the diagnostic pathway proved dynamic and prone to reclassification, underscoring that initial bedside differentiation between erysipelas, cellulitis, and abscess remains challenging in routine care. Imaging and microbiology reflected real-world practice: CT was more frequently used in patients with higher inflammatory markers and was associated descriptively with greater procedural burden and more re-interventions. These observations may reflect confounding by indication, but the retrospective design and absence of multivariable adjustment preclude determining whether CT use was independently associated with disease severity or operative management. Accordingly, these findings should be considered hypothesis-generating. When cultures were positive, pathogens were predominantly Gram-positive, supporting the frequent use of aminopenicillin/β-lactamase inhibitor regimens, while the notable use of broader-spectrum agents likely mirrors uncertainty and concern for deep or complicated disease. Overall, our findings highlight key pitfalls in extremity SSTI management—heterogeneous documentation, evolving diagnostic labels, and the difficulty of distinguishing uncomplicated from complicated cellulitis retrospectively—and support a structured approach that combines careful clinical reassessment with targeted imaging when anatomical clarification is likely to change management.

## Limitations

This study has several important limitations. First, its retrospective single-center design limits causal inference and generalizability and introduces selection bias toward patients requiring inpatient—and frequently surgical—management. Detailed comorbidities were retrospectively extracted from routine clinical documentation rather than systematically assessed according to predefined criteria. This may have resulted in underdocumentation of individual conditions, particularly chronic corticosteroid use and other forms of pharmacological immunosuppression. Accordingly, the observed frequency of conditions potentially affecting immune competence may underestimate the true prevalence in this cohort. Second, diagnostic labels (erysipelas, cellulitis, abscess) were based on routine clinical documentation and often changed during hospitalization; thus, misclassification is likely and cannot be fully corrected. Third, the key clinical distinction between uncomplicated vs. severe cellulitis might not have been operationalized reliably from the available records; consequently, severity had to be approximated using surrogates such as operative treatment and re-interventions, which may incompletely capture true disease extent. Furthermore, diagnosis-stratified analyses included small subgroups, particularly the isolated abscess group, resulting in imprecise estimates and limiting meaningful between-group comparisons. These subgroup findings should therefore be regarded as descriptive and exploratory. Fourth, associations between imaging modality and surgery may be subject to substantial confounding by indication (e.g., CT preferentially performed in patients with higher inflammatory markers or suspected deep extension), and the temporal sequence (imaging preceding vs. following the decision to operate) could not be reconstructed consistently. Fifth, the local center includes a dermatology department managing soft tissue infections that do not require surgical intervention. Consequently, case selection has shifted toward patients likely needing surgical management. Finally, discharge CRP and leukocyte values were missing in 19/242 (7.9%) patients. As missingness may not have been random, the observed decline in inflammatory markers may be biased and should be interpreted with caution.

## Data Availability

Data available on request due to privacy/ethical restrictions.
